# *Wisteria floribunda agglutinin*-binding glycan expression is decreased in endometriomata

**DOI:** 10.1186/1477-7827-12-100

**Published:** 2014-10-24

**Authors:** Tomoko Hirakawa, Kaei Nasu, Kentaro Kai, Yoko Aoyagi, Terukazu Ishii, Tetsuya Uemura, Mitsutake Yano, Hisashi Narahara

**Affiliations:** Department of Obstetrics and Gynecology, Faculty of Medicine, Oita University, Idaigaoka 1-1, Hasama-machi, Yufu-shi Oita, 879-5593 Japan; Division of Obstetrics and Gynecology, Support System for Community Medicine, Faculty of Medicine, Oita University, Idaigaoka 1-1, Hasama-machi, Yufu-shi Oita, 879-5593 Japan

**Keywords:** Endometriosis, *Wisteria floribunda agglutinin*, Lectin microarray, N-acetylgalactosaminyl transferase

## Abstract

**Background:**

Glycosylation is one of the most common post-translational modifications of eukaryotic proteins and is known to undergo dynamic changes in a wide range of biological processes. To date, however, the glycan expression profiles in endometriosis are largely unknown. The objective of the study was to identify the panel of glycans that were aberrantly expressed in endometriosis, a hormone-dependent disease.

**Methods:**

The glycan expression profiles in primary cultured human endometriotic cyst stromal cells (ECSCs) and normal endometrial stromal cells (NESCs) were determined by lectin microarray analysis. Distribution of *Wisteria floribunda agglutinin* (WFA)-binding glycans in ovarian endometriotic cysts and eutopic proliferative phase endometrium were assessed by lectin histochemistry. The expressions of N-acetylgalactosaminyl transferases that synthesize WFA-binding glycans were evaluated in ECSCs and NESCs.

**Results:**

We found that the levels of WFA-binding glycans were decreased in ECSCs. Lectin histochemistry revealed that WFA-binding glycans were decreased only in the stromal components of the ovarian endometriotic cysts, but not in the epithelial components, compared to the eutopic proliferative phase endometrium. The expressions of N-acetylgalactosaminyl transferases that synthesize WFA-binding glycans were downregulated in ECSCs.

**Conclusions:**

Utilizing lectin microarray analysis and lectin histochemistry, we found that WFA-binding glycans were decreased in endometriosis. The synthetic enzymes of WFA-binding glycans were significantly downregulated in ECSCs. It is suggested that reduced expression of N-glycans with WFA-binding properties on ECSCs is a novel characteristics of endometriosis.

## Background

Endometriosis is characterized by dysmenorrhea, chronic pelvic pain and/or subfertility, all of which can significantly impair the quality of life of young women, who comprise the population most likely to suffer from this estrogen-dependent disease [1]. Although endometriotic tissues and normal proliferative endometrium are histologically similar, several molecular differences between normal and endometriotic tissues have been identified [2–5].

In the field of reproductive medicine and oncology, glycan profiling is attracting attention because it can provide valuable new information about cells and tissues. It has been estimated that 1% of the human genome is involved in the production and modification of glycans [6]. Glycans are produced in a non-template-driven manner in different monosaccharides combine in a branched fashion and a variety of orientations, producing isomeric and isobaric glycans. Glycans have been shown to reflect cellular conditions during development, differentiation, adhesion, proliferation, aging, and activation, and, inflammation, infection, and tumorigenesis through specific binding to their glycan ligands [7–11]. It has also been estimated that as many as 70% of all human proteins are glycosylated [12]. As one of the most common post-translational modifications of eukaryotic proteins, glycosylation undergoes dynamic changes in a wide range of biological processes [13]. However, glycan profiling in endometriosis have not been elucidated yet, which encouraged us to carry out the present glycomic study.

Glycans are bound specifically by glycan-binding proteins (GBPs), or lectins, which are diverse families of ubiquitous proteins found in viruses, fungi, bacteria, plants, and animals (including humans) [14, 15]. The endogenous functions of GBPs/lectins have not been well established. Lectin microarrays, an emerging modern technology, allow the ultrasensitive detection of multiplex lectin–glycan interactions. These arrays have been used extensively in studies of pluripotent and somatic stem cells and carcinogenesis [16–23].

The present study was designed to identify the panel of glycans that were aberrantly expressed in primary cultured human endometriotic cyst stromal cells (ECSCs) in comparison with primary cultured human normal endometrial stromal cells (NESCs). Lectin microarray analysis revealed *Wisteria floribunda* agglutinin (WFA)-binding glycans were significantly decreased in ECSCs compared to NESCs. Thereafter, we focused on WFA and evaluated the distribution of WFA-binding glycans in the ovarian endometriotic cysts and to the eutopic proliferative phase endometrium by lectin histochemistry. The expressions of N-acetylgalactosaminyl transferases that synthesize WFA-binding glycans were also examined in ECSCs and NESCs. The present study may provide a platform for the future studies on aberrant glycan expressions in endometriosis.

## Methods

### Ethical approval

The present study was approved by the institutional review board of the Faculty of Medicine, Oita University, and signed informed consent was obtained from all patients (Reference No.: P-10-04; Date of approval: July 23, 2010). This study was conducted between September 1, 2013 and May 13, 2014.

### Tissue collection, ECSC and endometrial stromal cell (NESC) isolation procedure

Endometriotic tissues were obtained from premenopausal patients who had undergone a surgery for ovarian endometriotic cysts (n = 35 in total, aged 26–37 years). Normal endometrial tissues were obtained from premenopausal patients who had undergone hysterectomies for subserosal leiomyoma and had no evidence of endometriosis (n = 39 in total, aged 35–41 years). None of the patients had received any hormonal treatments for at least 2 years prior to the operation. All of the specimens were diagnosed as being in the mid- to late-proliferative phases, using a standard histological examination of the endometrial tissues.

Of these collected samples, sixteen endometriotic tissues and twenty normal endometrial tissues were fixed in 7% neutral buffered formaldehyde solution, embedded in paraffin, and processed for lectin histochemical staining [24].

Whereas, other 19 ovarian endometriotic tissues and 19 normal eutopic endometrial tissues were processed for cell isolation, as previously described [25, 26]. Briefly, the tissues were minced in Hank’s balanced salt solution and digested with 0.5% collagenase (Gibco-BRL, Gaithersburg, MD, USA) in Dulbecco’s modified Eagle’s medium (DMEM) (Gibco-BRL) at 37°C for 40 min. The dispersed cells were filtered through a 70-μm nylon mesh to remove the undigested tissue pieces. The filtrated fraction was further separated from epithelial cell clumps by differential sedimentation at unit gravity as follows. The cells were resuspended in 2 ml of culture medium and layered slowly over 10 ml of the medium in a centrifuge tube. Sealed tubes were placed in an upright position at 37°C in 5% CO_2_ in air for 30 min. After sedimentation, the top 8 ml of the medium was collected. Finally, the medium containing stromal cells was filtered through a 40-μm nylon mesh.

Final purification was achieved by allowing stromal cells, which rapidly attached to plates, to adhere selectively to culture dishes for 30 min at 37°C, followed by the removal of non-adhering epithelial cells. Isolated ECSCs and NESCs were cultured in DMEM supplemented with 100 IU/ml of penicillin (Gibco-BRL), 50 mg/ml of streptomycin (Gibco-BRL) and 10% heat-inactivated fetal bovine serum (FBS) (Gibco-BRL) at 37°C in 5% CO2 in air. ECSCs and NESCs in monolayer cultures after the third passage were >99% pure as analysed by immunocytochemical staining with antibodies against vimentin (V9; Dako, Copenhagen, Denmark), CD10 (SS2/36; Dako), cytokeratin (Dako), factor VIII (Dako) and leukocyte common antigen (2B11 t PD7/26, Dako) and were used for the following experiments [25, 26]. The cells separately isolated from individual patients were used in each experiment.

### Protein extraction for lectin microarray

Cultured ECSCs (n = 9) and NESCs (n = 11) were harvested by trypsinization. The cells were pelleted by washing with phosphate-buffered saline (PBS) by centrifugation, then solubilized with 20 μl of PBS containing 0.5% Nonidet P40, and sonicated gently. The cell suspensions were incubated on ice for 60 minutes and centrifuged at 20,000 *g* for 5 min at 4°C. The obtained supernatants were used as detergent-solubilized glycoprotein extract.

### Lectin microarray analysis

Lectin microarray analysis was performed according to the manufacturer’s instruction. Briefly, each 20 μl aliquot of the above glycoprotein solutions was incubated with 10 μg of Cy3-succimidyl ester (Amersham Biosciences, Tokyo, Japan) for 60 minutes at room temperature in the dark. The obtained Cy3-labeled glycoprotein was subjected to the lectin chip (LecChipTM; GP Biosciences, Yokohama, Japan) that contain 45 lectins for human cells. One hundred microliters of Cy3-labeled glycoprotein solution in probing buffer (Tris-buffered saline containing 0.05% Triton X-100) were applied to each well with immobilized lectins, at a concentration of 125 ng/ml. Incubation was performed at 4°C for about 15 hours until the binding reached equilibrium. After the incubation, we acquired a fluorescence image of the array using an evanescent-field fluorescence scanner, GlycoStation™ Reader 1200, SC-Profiler (GP BioScience). We calculated the net intensity value for each spot by subtracting a background value from signal intensity and averaged the signal net intensity values of three spots. To calculate the fold change of each lectin-binding glycans in ECSCs (n = 9) relative to NESCs (n = 11), the data for a given lectin were normalized to the median of that lectin in the eleven NESCs. Among 45 lectins, differentially expressed lectins were statistically extracted by a fold change of >2.0 combined with two-sided Mann–Whitney *U* test, with the Benjamini-Hochberg adjustment for false discovery rate (FDR) (*p* < 0.05).

### Lectin histochemistry

Lectin histochemical staining using streptavidin-biotin-peroxidase method was performed with biotinylated WFA (B-1355, Vector Laboratories, Peterborough, UK). Dewaxed and hydrated sections of ovarian endometriotic cyst (n = 16) and proliferative phase eutopic endometrium (n = 20) were immersed in 10 mM citric acid (pH 6.0) and autoclaved at 120°C for 10 min. Antigen activated sections were immersed in 0.3% hydrogen peroxide in methanol at room temperature for 10 min to block endogenous peroxidase activity, rinsed, microwaved in ethylenediaminetetraacetic acid buffer (1 mM, pH 8.0) for 10 min and equilibrated in PBS. Sections were blocked with 1% bovine serum albumin at room temperature for 10 min, before overnight incubation in 10 μg/ml biotinylated WFA at room temperature for 150 min. The sections were rinsed in PBS before incubation with Streptavidin-peroxidase reagent (424021, Histofine simple stain SAB-PO (M) kit, Nichirei Co., Tokyo, Japan) at room temperature for 5 min. Subsequently, the sections were rinsed in distilled water before 60 sec-incubation with 3,3’-diaminobenzidine tetra hydrochloride (Histofine DAB substrate kit, Nichirei Co.). Sections were washed in distilled water, counterstained with hematoxylin, dehydrated, cleared and mounted. Histochemical staining score was used to assess the staining intensity with light microscopy (Zeiss Axiophot; Carl Zeiss, Oberkochen, Germany).

### Total RNA isolation and quantitative reverse transcription-polymerase chain reaction (RT-PCR) for N-acetylgalactosaminyl transferases

WFA binds with high affinity to glycans containing N-acetyl-D-galactosamine (GalNAc), namely GalNAcb1-3Gal, GalNAcb1-4Gal, and GalNAcb1-4GlcNAc [27]. Whereas, the cognate glycosyltransferases of WFA include β1,3-N-acetylgalactosaminyl transferase (B3GALNT) 1, B3GALNT2, β1,4-N-acetylgalactosaminyltransferase (B4GALNT) 1, B4GALNT2, B4GALNT3, and B4GALNT4 [28]. Therefore, we next evaluated the expression of these N-acetylgalactosaminyl transferases in ECSCs and NESCs.

Total RNA from cultured ECSCs (n = 7) and NESCs (n = 5) was extracted with a RNeasy Mini kit (Qiagen, Valencia, CA, USA). The quality of the extracted RNA was confirmed by measuring the absorbance at 230 nm, 260 nm, and 280 nm using a spectrophotometer (NanoDrop 2000, Thermo Scientific, Wilmington, DE, USA), and subjected to RT-PCR as described previously [24].

Quantitative RT-PCR was carried out in with a LightCycler 480 (Roche Diagnostics, Penzberg, Germany) using the TaqMan Universal PCR Master Mix II No AmpErase UNG (Applied Biosystems) with B3GALNT1-specific (Assay ID: Hs00364202_s1, Applied Biosystems, Carlsbad, CA, USA), B3GALNT2-specific (Assay ID: Hs00380823_m1, Applied Biosystems), B4GALNT1-specific (Assay ID: Hs00155195_m1, Applied Biosystems), B4GALNT2-specific (Assay ID: Hs00396440_m1, Applied Biosystems), B4GALNT3-specific (Assay ID: Hs00419636_m1, Applied Biosystems), B4GALNT4-specific (Assay ID: Hs00331790_m1, Applied Biosystems) or glyceraldehyde 3-phosphate dehydrogenase (GAPDH)-specific (Assay ID: Hs02758991_g1, Applied Biosystems) primers [24]. Data from the RT-PCR experiments are presented as the percentage of each N-acetylgalactosaminyl transferase/GAPDH of ECSCs to those of NESCs.

### Western blot analysis

The protein levels of N-acetylgalactosaminyl transferases in ECSCs (n = 3) and NESCs (n = 3) were investigated by Western blot analysis [24]. Antibodies against B3GALNT1 (ab98873, Abcam, Tokyo, Japan), B3GALNT2 (ab156785, Abcam), B4GALNT1 (ab55065, Abcam), B4GALNT2 (ab176856, Abcam), B4GALNT3 (ab82800, Abcam), B4GALNT4 (sc-166982, Santa Cruz Biotechnology, CA, USA) and GAPDH (Ambion, Austin, TX, USA) were used as primary antibodies. The relative expression of each N-acetylgalactosaminyl transferase protein in ECSCs and NESCs was analyzed using Image Lab™ software (Bio-Rad Laboratories, Hercules, CA, USA).

### Statistical analysis

Data were calculated as percentages to the corresponding controls, presented as means ± SD, and were appropriately analyzed by the two-sided Mann–Whitney *U* test, the Student’s *t* test, or Bonferroni test with Sigmaplot 11.2 (Systat Software, San Jose, CA, USA). Values of *p* < 0.05 were considered statistically significant.

## Results

### Identification of glycans differentially expressed in ECSCs and NESCs

**Figure 1:**
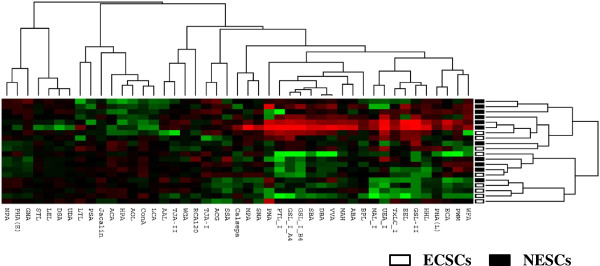
**Unsupervised hierarchical clustering of ECSCs and NESCs.** ECSCs (n = 9) and NESCs (n = 11) were clustered according to the glycan expression profiles with 45 lectin probes that meet the filtering criteria. Unsupervised hierarchical clustering analysis of these 45 lectins showed that the patterns of glycan expression in ECSCs were similar to those in NESCs as a whole, suggesting that diseased tissues in endometriosis retain the characteristics of their origin, namely the eutopic endometrium, concerning glycan expression profiles.

We used a lectin microarray platform covering a total of 45 human lectins to determine the expression patterns of glycans in ECSCs (n = 9) and NESCs (n = 11). Unsupervised hierarchical cluster analysis of these 45 lectins showed that the patterns of glycan expression in ECSCs were similar to those in NESCs as a whole (Figure 1), suggesting that diseased tissues in endometriosis retain the characteristics of their origin, namely the eutopic endometrium, concerning lectin-binding glycan expression profiles.

To identify the group of glycans that are differentially expressed between ECSCs and NESCs, we chose lectin-binding glycans with a fold change of >2.0 from 45 lectins. As shown in Table 1, we found that only WFA-binding glycans were significantly downregulated in ECSCs by using the two-sided Mann–Whitney *U* test and adjusted FDR of <0.05. Whereas, none of the lectin-binding glycans were found to be statistically upregulated in ECSCs compared to NESCs.

**Table 1:** **The lectin signals between ECSCs (n = 9) and NESCs (n = 11) obtained from a lectin microarray analysis**

Lectin	ECSC signals	NESC signals	ECSC/NESC	FDR-adjusted
	(Mean ± SD)	(Mean ± SD)	Signal ratio	***p***-value
GSL-II	2.9111 ± 2.685	12.773 ± 21.907	0.228	0.506
WFA	8.578 ± 4.499	23.691 ± 10.474	0.362	0.031
EEL	10.422 ± 7.788	24.227 ± 33.06	0.430	0.442
GSL_I_A4	14.5 ± 14.009	33.018 ± 51.382	0.439	0.459
GSL_I_B4	12.344 ± 12.107	27.973 ± 33.193	0.441	0.481
VVA	11.122 ± 7.198	24.918 ± 22.587	0.446	0.596
ECA	9.722 ± 5.908	19.2 ± 17.569	0.506	0.59
UEA_I	6.033 ± 5.96	11.909 ± 14.76	0.507	0.486
DBA	12.133 ± 8.693	23.536 ± 21.247	0.516	0.521
PTL_I	12.2 ± 12.806	22.136 ± 33.735	0.551	0.508
SBA	13.178 ± 10.764	23.7 ± 20.775	0.556	0.571
HHL	17 ± 9.687	30.473 ± 28.645	0.558	0.541
PWM	9.744 ± 5.585	17.182 ± 9.881	0.567	0.45
MAH	16.8 ± 5.232	28.836 ± 27.646	0.583	0.464
ABA	90.233 ± 22.797	143.064 ± 120.478	0.631	0.483
PNA	4.967 ± 4.438	7.791 ± 8.819	0.637	0.501
MPA	15.444 ± 4.319	22.818 ± 30.326	0.677	0.554
TxLC_I	17.422 ± 9.327	24.845 ± 19.056	0.701	0.492
ACG	64.922 ± 34.0556	92.445 ± 37.676	0.702	0.585
MAL_I	18.056 ± 12.372	24.864 ± 22.1	0.726	0.511
BPL	25.8 ± 7.434	34.618 ± 15.621	0.745	0.533
SNA	33.144 ± 13.886	44.355 ± 34.96	0.747	0.488
SSA	36.811 ± 15.962	48.909 ± 27.953	0.753	0.495
PHA(L)	12.533 ± 5.879	16.264 ± 6.687	0.771	0.453
RCA120	66.022 ± 16.372	82.918 ± 31.243	0.796	0.528
TJA-II	37.367 ± 15.26	45.945 ± 12.496	0.813	0.461
GNA	93.978 ± 23.352	109.018 ± 43.165	0.862	0.504
WGA	98.011 ± 25.708	112.455 ± 31.601	0.872	0.475
TJA-I	49.278 ± 24.915	55.745 ± 16.598	0.884	0.611
AAL	52.178 ± 18.876	58.445 ± 20.733	0.893	0.595
Calsepa	45.089 ± 15.359	49.445 ± 28.858	0.912	0.775
Jacalin	168.8 ± 35.876	172.218 ± 42.136	0.98	0.847
STL	679.567 ± 70.057	656.364 ± 168.174	1.0354	0.77
NPA	179.722 ± 54.043	172.7 ± 50.535	1.0407	0.787
PHA(E)	146.678 ± 36.955	139.018 ± 49.862	1.055	0.766
ACA	145.689 ± 51.242	136.455 ± 66.262	1.068	0.763
LTL	33.667 ± 13.814	31.255 ± 16.35	1.077	0.777
LEL	548 ± 50.485	508.155 ± 117.768	1.078	0.508
UDA	313.967 ± 37.292	285.464 ± 71.134	1.1	0.502
DSA	443.7 ± 77.582	396.864 ± 132.835	1.118	0.494
HPA	89.722 ± 26.453	73.982 ± 41.287	1.213	0.509
PSA	85.311 ± 14.361	66.655 ± 22.578	1.28	0.433
AOL	91.611 ± 17.239	70.855 ± 24.037	1.293	0.568
LCA	263.311 ± 42.57	198.2 ± 70.68	1.329	0.477
ConA	393.767 ± 112.746	294.991 ± 141.82	1.335	0.642

FDR, false discovery rate.

### Distribution of WFA-binding glycans in ovarian endometriotic cysts and normal eutopic endometrium

**Figure 2:**
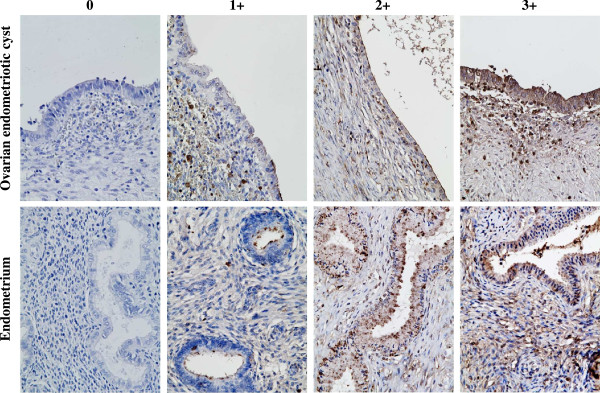
**Distribution of WFA-binding glycans in ovarian endometriotic cysts and normal proliferative phase endometrium.** Both the glandular epithelial cells and the stromal component of ovarian endometriotic tissues as well as the normal proliferative phase endometrium were positive for WFA-binding glycans with strong to weak intensity depending on the samples (hematoxylin & eosin staining, original magnification ×40). Staining intensity for WFA-binding glycans assessed by histochemical staining score is summarized in Table 2.

We focused on WFA for the following experiments. Both the glandular epithelial cells and the stromal component of ovarian endometriotic cysts as well as the normal proliferative phase endometrium were positive for WFA-binding glycans with strong to weak intensity (Figure 2 and Table 2). Significantly decreased staining for WFA-binding glycans was observed in endometriotic stromal component in comparison with eutopic endometrium. Whereas, similar staining patterns were observed in the epithelial component in these tissues.

**Table 2:** **WFA-staining scores in ovarian endometriotic cysts (n = 16) and proliferative-phase eutopic endometrium samples (n = 20)**

	Epithelial component		Stromal component	
WFA-staining score	Endometriotic cyst	Eutopic endometrium	Endometriotic cyst	Eutopic endometrium
3	7	12	3	9
2	6	5	5	7
1	3	3	7	4
0	0	0	1	0
Mean ± SD	2.3 ± 0.8	2.5 ± 0.8	1.6 ± 0.9*	2.3 ± 0.8

The WFA-staining intensity was evaluated separately for the epithelial component and the stromal component and stratified as follows: 0 (negative), 1 (weak), 2 (moderate), and 3 (strong). Two gynecologists (K. K. and M. Y.) scored all samples independently, and discrepancies were resolved by simultaneous examination. *p < 0.05 vs. eutopic endometrium (Student’s *t*-test).

### Messenger RNA (mRNA) expression of N-acetylgalactosaminyl transferases in ECSCs and NESCs

**Figure 3:**
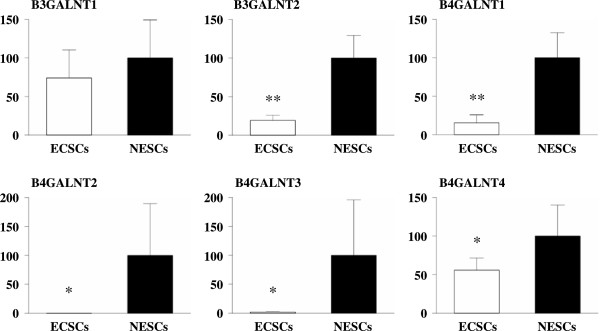
**mRNA expression of N-acetylgalactosaminyl transferases in ECSCs and NESCs.** The mRNA expressions of B3GALNT1, B3GALNT2, B4GALNT1, B4GALNT2, B4GALNT3, and B4GALNT4 in ECSCs (n = 7) and NESCs (n = 5) were evaluated by quantitative RT-PCR. *p < 0.025 and **p < 0.0001vs. NESCs (Student’s *t* test).

The mRNA expressions of B3GALNT1, B3GALNT2, B4GALNT1, B4GALNT2, B4GALNT3, and B4GALNT4 in ECSCs and NESCs were evaluated by quantitative RT-PCR. As shown in Figure 3, the mRNA expressions of B3GALNT2, B4GALNT1, B4GALNT2, B4GALNT3, and B4GALNT4 in ECSCs were significantly lower than those in NESCs. Although there was no significance, the mRNA expression of B3GALNT1 tends to decrease in ECSCs compared to NESCs.

### Protein levels of N-acetylgalactosaminyl transferases in ECSCs and NESCs

**Figure 4:**
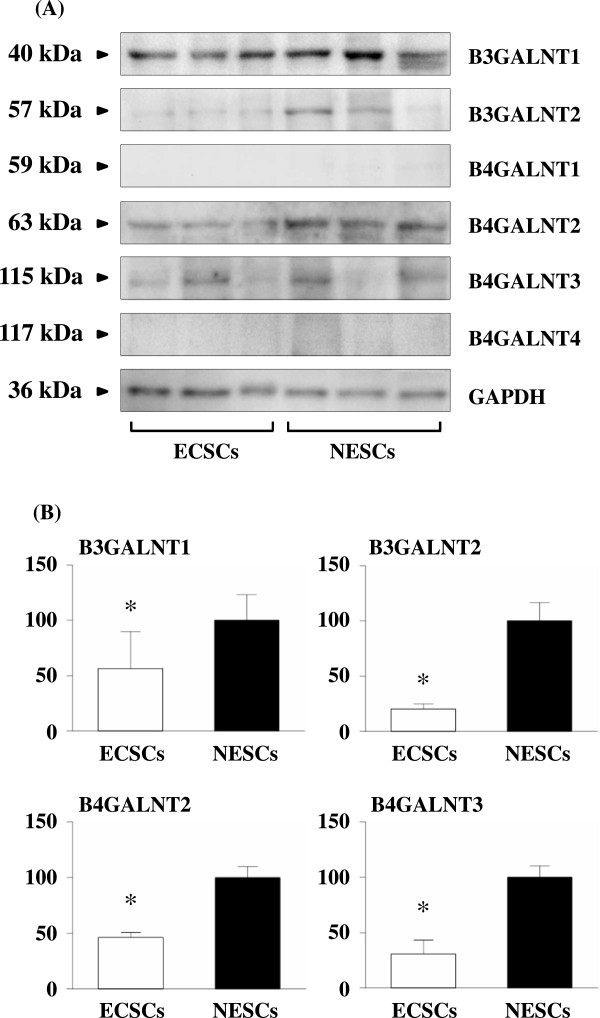
**Protein levels of N-acetylgalactosaminyl transferases in ECSCs and NESCs. (A)** Results of Western blot analysis. **(B)** Relative protein levels of N-acetylgalactosaminyl transferases in ECSCs (n=3) and NESCs (n=3). The levels of B3GALNT1, B3GALNT2, B4GALNT2, and B4GALNT3 were significant higher in NESCs compared to ECSCs. Whereas, the protein expressions of B4GALNT1 and B4GALNT4 in ECSCs and NESCs were under the detection levels. *p < 0.005 vs. NESCs (Student’s *t* test).

The protein expressions of B3GALNT1, B3GALNT2, B4GALNT1, B4GALNT2, B4GALNT3, and B4GALNT4 in ECSCs and NESCs were evaluated by Western blot analysis. As shown in Figure 4, the protein expressions of B3GALNT1, B3GALNT2, B4GALNT2, and B4GALNT3 were detected in ECSCs and NESCs. The levels of these four N-acetylgalactosaminyl transferases were significant higher in NESCs compared to ECSCs. Whereas, the protein expressions of B4GALNT1 and B4GALNT4 in ECSCs and NESCs were under the detection levels.

## Discussion

The current study was conducted to identify the panel of aberrantly expressed glycans in ECSCs in comparison with those in NESCs. Utilizing the lectin microarray technique, we found, for the first time, that WFA-binding glycans were decreased in ECSCs. Lectin histochemistry revealed that WFA-binding glycans were decreased only in the stromal components of the ovarian endometriotic cysts, but not in the epithelial components, compared to the eutopic proliferative phase endometrium. Thereafter, we focused on the synthetic enzymes of WFA-binding glycans and designed further experiments. The mRNA levels of B3GALNT2, B4GALNT1, B4GALNT2, B4GALNT3, and B4GALNT4 were significantly downregulated in ECSCs, whereas the protein levels of B3GALNT1, B3GALNT2, B4GALNT2, and B4GALNT3 were significantly downregulated in ECSCs. The meanings of aberrant glycan expressions in endometriosis are not elucidated from the present study, however, it is speculated that aberrantly expressed glycans in ECSCs may involve in the development of disease-specific features of endometriosis. The present findings may provide a platform for the future glycomic studies in endometriosis.

A huge number of possible unique sequences can be composed of glycans, from basic building units. Glycans are thus excellent information-coding “tools”. Cummings [29] estimated that up to 500,000 glycan-modified biomolecules may form a cellular glycome, based on 7,000 unique glycan sequences. Arnaud et al. [30] estimated that in humans, 70% of proteins and (80% of membrane-bound proteins) are glycosylated. Indeed, glycosylation is a very common type of post-translational modification of proteins, with important roles in a wide range of cellular events. Cell development, differentiation, morphogenesis, proliferation, adhesion, fertilization, embryogenesis, immunity, infection, and tumorigenesis have all been shown to involve protein glycosylation [7–11, 31–33].

Oncology researchers have studied aberrant glycosylation extensively. For example, the invasion and metastatic potential of cancer cells were found to be strongly correlated cell surface sialylation and the β1–6 branching of N-glycans [33–36]. The heavily O-glycosylated tumor-associated mucin known as sialylated mucin 1 [37] expresses Tn (GalNAc) antigens and T (Galb1, 3GalNAc) antigens that are linked to cellular adhesion and functioning of the immune system [38, 39]. In addition, several glycan-based biomarkers have been established, including α-fetoprotein 1 [40], prostate-specific antigen [41], the carcino-embryonic antigen family [42, 43], and cancer antigen 19–9 [44]. The complete array of glycans associated with endometriosis has not been determined.

Lectins each have a unique specificity profile, and the differing specificities for complex glycans make lectins a valuable tool for identifying a particular type of glycan. Together the lectins comprise a diverse group of carbohydrate-binding proteins [10]. Lectin microarrays are used to profile the many types of glycan structures observed at the cell surface and in glycoconjugates [10]. Berkes et al. [45] evaluated the plasma N-glycan levels in endometriosis patients by hydrophilic interaction high performance liquid chromatography and found the significant decrease of some of the glycan peaks. In the present study, we used a recently developed lectin microarray that consists of 45 lectins with different binding preferences covering *N-* and *O*-linked glycans [23] and we found that WFA-binding glycans were decreased in ECSCs.

The reactivity of WFA is well-documented in mammary glands [46], cholangiocarcinoma [16, 17], prostate cancer [47], and the central nervous system [48]. WFA reactivity was shown to be upregulated in prostate cancer [47] and cholangiocarcinoma [17]. Matsuda et al. [17] demonstrated that WFA reactivity was associated with MUC1 in cholangiocarcinoma. WFA specifically recognizes terminal α/β-N-acetylgalactosamine residues on N-glycans [28, 46, 49].

The genes B3GALNT1, B3GALNT2, B4GALNT2, and B4GALNT3 synthesize WFA-binding glycans [28], and we found that their expressions were significantly downregulated in ECSCs, a finding that is consistent with the present lectin microarray and lectin histochemistry results. In some tumors, glycosyltransferase expression levels were shown to be up- or down-regulated [50–53]. We did not evaluate which glycoproteins were affected by these decreased N-acetylgalactosaminyl transferases in the present study. Further detailed examinations of this matter are needed to elucidate the importance of aberrant glycan expression in the pathogenesis of endometriosis.

To our knowledge, hormonal regulation of glycan expressions in the normal cyclic endometrium as well as the diseased endometrium has not been elucidated yet. Whereas, we did not evaluate the glycan profiles in the eutopic endometrium of the endometriosis patients in the present study. These are the limitations of the present study and we would like to elucidate these points in the future.

## Conclusions

Utilizing lectin microarray analysis and lectin histochemistry, we found that WFA-binding glycans were decreased in ECSCs. The synthetic enzymes of WFA-binding glycans were significantly downregulated in ECSCs. It is suggested that reduced expression of N-glycans with WFA-binding properties on ECSCs is a novel characteristics of endometriosis. Further studies on the repertoire of glycomics may provide useful information on the pathogenesis of endometriosis.

## Abbreviations

B3GALNT: β1,3-N-acetylgalactosaminyl transferase
B4GALNT: β1,4-N-acetylgalactosaminyltransferase
ECSCs: Endometriotic cyst stromal cells
FDR: False discovery rate
GalNAc: N-acetyl-D-galactosamine
GAPDH: Glyceraldehyde 3-phosphate dehydrogenase
GBPs: Glycan-binding proteins
mRNA: Messenger RNA
NESCs: Normal endometrial stromal cells
PBS: Phosphate-buffered saline
RT-PCR: Reverse transcription-polymerase chain reaction
WFA: *Wisteria floribunda agglutinin*.
